# Special Issue: New Approaches to Counteract Drug Resistance in Cancer

**DOI:** 10.3390/molecules22010006

**Published:** 2016-12-23

**Authors:** M. Helena Vasconcelos

**Affiliations:** 1 i3S—Instituto de Investigação e Inovação em Saúde da Universidade do Porto, Rua Alfredo Allen 208, Porto 4200-135, Portugal; hvasconcelos@ipatimup.pt; Tel.: +351-220-408-800; Fax: +351-225-570-799; 2 Cancer Drug Resistance Group, IPATIMUP—Institute of Molecular Pathology and Immunology of the University of Porto, Porto 4200-135, Portugal; 3 Department of Biological Sciences, FFUP—Faculty of Pharmacy of the University of Porto, Rua de Jorge Viterbo Ferreira 228, Porto 4050-313, Portugal

Drug resistance is a major impediment to the successful treatment of cancer patients. Resistance might be intrinsic (when occurring from diagnosis) or acquired (when it develops during the course of treatment). Acquired drug resistance is most likely due to the selective pressure caused by drug treatment or radiation treatment [1]. This clinical problem is very difficult to resolve due to several factors, such as intratumor genetic heterogeneity and tumor dynamics [2] together with the presence of cancer stem cells [3]. The mechanisms responsible for the drug resistant phenotype depend on the source of the tumor cells and on the mechanism of action of the drug [1]. Moreover, often tumors are multidrug resistant (MDR), i.e., they are resistant to several drugs that have different molecular targets and different chemical structures [4].

This Special Issue of Molecules intended to collect state-of-the-art original research and review articles on new approaches to counteract drug resistance in cancer. The papers published include two review articles and seven original research articles.

The two review articles highlight two important aspects in current research regarding drug resistance. The review by Du B and Shim J.S. [5], focuses on the increasingly recognized role of epithelial-mesenchymal transition (EMT) in cancer drug resistance. Indeed, the role of EMT in decreased cell adhesion and in the increased potential for cell motility, invasion, and metastasis has been long documented, but more recently it has been shown that the differentiation state of tumor cells also contributes to acquired drug resistance [6]. The review published in this Special Issue of Molecules [5] aimed to describe the known mechanisms involved in the relationship between EMT and drug resistance, while emphasizing the new possibilities that targeting EMT might bring to overcoming cancer drug resistance. This work is of instrumental relevance to the field of cancer drug target development.

The review written by Long S. et al. is a comprehensive review of marine natural products and derivatives (alkaloids, polyoxygenated sterols, polyketides, terpenoids, diketopiperazines, and peptides) with P-glycoprotein (P-gp) inhibitory activity [7]. An overexpression of ATP-binding cassette (ABC) transporters, including P-gp (also known as MDR1 or ABCB1), is responsible for many tumors becoming MDR, since these pumps cause drug efflux thereby decreasing intracellular drug concentrations in tumor cells [4,8,9]. This review not only presents the synthetic pathways for the most promising P-gp inhibitor marine compounds, but also highlights their established structure-activity relationships, making this an invaluable source of information for medicinal chemists aiming to develop novel drug candidates based on natural products to counteract drug resistance.

Several of the research papers in this Special Issue refer to work developed in order to identify novel P-gp inhibitors. Pan G. et al. verified that Alisol F 24 acetate (a triterpene extracted from the dry tubers of *Rhizoma alismatis*) could reverse the MDR phenotype of a multidrug resistant human breast cancer cell line, by inhibiting P-gp-mediated drug efflux, while having no effect on the drug sensitive counterpart cells [10]. In another work, Zhang X.Y. et al. showed that osimertinib (AZD9291), a clinically-approved third-generation EGFR (epidermal growth factor receptor) tyrosine kinase inhibitor, inhibited P-gp-mediated drug efflux and stimulated its ATPase activity, significantly sensitizing ABCB1-transfected and drug-selected cell lines to colchicine, paclitaxel, and vincristine [11]. Additionally, the paper from Yuan F. et al. showed that extracts from *Periplaneta americana* reversed MDR in a hepatocellular carcinoma cell line, by mechanisms which included the inhibition of expression of three multidrug resistance-associated proteins: P-gp, drug resistance-associated protein (MRP), and lung resistance-related protein (LRP) [12]. These papers suggest new potential therapeutic strategies to overcome MDR.

The horizontal transfer of drug resistance, mediated by extracellular vesicles (EVs) such as exosomes or microvesicles, has been documented in several cancer cell models. This intercellular transfer of drug resistant traits from cancer drug resistant (donor cells) to drug sensitive (recipient cells) is mediated by the cargo of the EVs released by the donor cells, which may contain drug-efflux pumps, miRNAs, long noncoding RNAs, and other mediators [13]. Following a previously described proposal for the involvement of a unique protein complex in regulating the intercellular transfer of P-gp by EVs [14], Pokharel D. et al. showed in this Special Issue that these mediators (Ezrin, Radixin, and Moesin, together with CD44) have a role in the regulation of P-gp functionality and in the acquisition of MDR by the recipient cells. This study is highly relevant since it identifies candidate proteins as potential new therapeutic targets to overcome MDR [15].

The discovery of novel compounds with antitumour activity and chemosensitizing effects may in the long term contribute to overcoming drug resistance. The paper of Vedarethinam V. et al. described the antitumor and apoptosis-inducing effect of synthetic Mannich base (1,3-bis-((3-hydroxynaphthalen-2-yl)phenylmethyl)urea) on hepatocellular carcinoma cells and in diethylnitrosamine-induced hepatocarcinoma in albino rats [16]. In another paper, Fonseca J. et al. showed that prenylated chalcone 2 is an antimitotic agent, causing mitotic spindle damage. In addition, this compound enhances the chemosensitivity of breast and lung cancer tumor cells to paclitaxel [17]. These papers contribute to identifying the effects of compounds with antitumor potential.

Finally, the development of in vivo cancel models that more accurately resemble the drug resistance and invasion properties of human cancers will contribute to overcoming drug resistance. The paper written by Stojković S. et al. evaluated the invasiveness of RC6 rat glioma cells both in vitro and in a new orthotopic animal model. This very interesting study shows that the development of chemoresistance induced an invasive phenotype of cancer cells. In addition, this work highlights the relevance of this orthotopic allograft model for studying new approaches to counteract drug resistance [18].

This Special Issue brings together reviews and original research papers which contribute to the growing knowledge on drug resistance and how to overcome this problem.
